# Supplementary material for: In situ protein micro-crystal fabrication by cryo-FIB for electron diffraction
Source: Biophys Rep. 2018 Nov 14;4(6):339–47. doi: 10.1007/s41048-018-0075-x (PMC6276065; doi:10.1007/s41048-018-0075-x)
Supplement: Supplementary file 2 — Supplementary material 1 (PDF 58 kb) [file 41048_2018_75_MOESM2_ESM.pdf]

Supplementary information

A SerialEM script to perform automatic collection of electron diffraction images from a continuously tilted crystal. (X denotes the starting angle, and Y denotes the end angle. Delay 1 means the interval time between each diffraction. 0.2 indicates that each rotation increases by 0.2°).

```
=========================
Start_angle = X
End_angle = Y
Angle = $start_angle
Loop 1000
TiltTo $angle
Delay 1
ReportTiltAngle
R
S
Angle = $angle + 0.2
If $angle > $end_angle
    break
Endif
EndLoop
=========================
```
